# Past, Present and (Foreseeable) Future of Biological Anti-TNF Alpha Therapy

**DOI:** 10.3390/jcm12041630

**Published:** 2023-02-17

**Authors:** Gian Marco Leone, Katia Mangano, Maria Cristina Petralia, Ferdinando Nicoletti, Paolo Fagone

**Affiliations:** 1 Department of Biomedical and Biotechnological Sciences, University of Catania, Via S. Sofia 97, 95123 Catania, Italy; 2 Department of Clinical and Experimental Medicine, University of Messina, 98122 Messina, Italy

**Keywords:** monoclonal antibody, TNF-α, TNF-α inhibitors, biomarker, biosimilar agents, inflammation, COVID-19

## Abstract

Due to the key role of tumor necrosis factor-alpha (TNF-α) in the pathogenesis of immunoinflammatory diseases, TNF-α inhibitors have been successfully developed and used in the clinical treatment of autoimmune disorders. Currently, five anti-TNF-α drugs have been approved: infliximab, adalimumab, golimumab, certolizumab pegol and etanercept. Anti-TNF-α biosimilars are also available for clinical use. Here, we will review the historical development as well as the present and potential future applications of anti-TNF-α therapies, which have led to major improvements for patients with several autoimmune diseases, such as rheumatoid arthritis (RA), ankylosing spondylitis (AS), Crohn’s disease (CD), ulcerative colitis (UC), psoriasis (PS) and chronic endogenous uveitis. Other therapeutic areas are under evaluation, including viral infections, e.g., COVID-19, as well as chronic neuropsychiatric disorders and certain forms of cancer. The search for biomarkers able to predict responsiveness to anti-TNF-α drugs is also discussed.

## 1. Introduction: History of Biologics

One of the main breakthroughs of the past 50 years in medicine has been the development of biologics. The invention of hybridoma technology goes back to 1975, when Kohler and Milstein [1] developed this method to produce antigen-specific monoclonal antibodies (mAbs) by hybridizing B cells from immunized animals with immortalized mouse myeloma cells [1].

mAbs may exert two types of therapeutic effects: first, by binding an antigen, they can neutralize its biological functions; second, by opsonizing target cells, they may induce an effective Fc receptor-mediated immune response.

In 1986, the first murine mAB (muromonab CD-3) was approved by the USA Food and Drug Administration (FDA) for preventing acute kidney transplant rejection [2]. This drug selectively binds to the ε chain of the TCR-CD3 complex expressed on the surface of T lymphocytes, blocking their proliferation and differentiation and consequently leading to an immunodepression state. Although this was the first step toward the introduction of mAbs in clinical use, hybridoma technology exhibited several disadvantages. In particular, patients treated with murine mAbs produced a neutralizing anti-mouse antibody that reduced the efficacy of the mAbs and accelerated their clearance [3]. The overcoming of this hindrance was the development of genetic engineering techniques through which chimeric murine–human mAbs were generated [4]. In particular, in 1984, Boulianne and colleagues developed chimeric mAbs by fusing the murine variable domain of antigen-binding fragment (Fab) regions with human μ and κ domains of the fragment crystallizable (Fc) region [4]. In 1994, the first chimeric mAb (abciximab) received FDA approval as an antiplatelet therapy in patients with cardiovascular diseases [5], and in 1997, the anti-CD20 mAb, rituximab, was approved by the FDA to treat patients with non-Hodgkin lymphoma [6]. Then, abciximab and rituximab were also approved by the European Medicine Agency (EMA) in 1997 and 1998, respectively [7,8]. On the other hand, infliximab (Remicade) was the first chimeric anti-TNF-α mAb for the treatment of Chron’s disease [9]. The use of Remicade was then extended to other therapeutic areas, including RA (in combination with methotrexate), ankylosing spondylitis, psoriatic arthritis, ulcerative colitis, pediatric Crohn’s disease, plaque psoriasis and pediatric ulcerative colitis.

A further and highly significant improvement in the pharmacology of mAbs was achieved with the production of humanized mAbs that were developed by transplanting murine hypervariable segments of the complementary-determining regions (CDRs) into a human Ab structure [10,11,12]. In 1997, the anti-IL-2 receptor (daclizumab) was the first humanized mAb that received FDA approval for preventing renal transplant rejection [13]. Then, it was also approved by the EMA in 1999 [14]. Humanized anti-TNF-α mAbs include adalimumab, golimumab and certolizumab. Humanized mAbs are less immunogenic than murine and chimeric mAbs [11]. However, humanized mAbs still showed some limits. In fact, their production was hard work and expensive. In order to bypass these limitations, fully human mAbs were developed in the early 1990s, using display technologies (phage display and yeast display libraries) [15,16,17].

Another innovative approach to obtain fully human mAbs is based on transgenic animals that are genetically modified by inserting human immunoglobulin genes into their genome sequence deprived of immunoglobulin loci. Hence, plasma B cells of transgenic animals can produce fully human mAbs after exposure to a specific antigen [18,19]. Although this technology was developed in 1994, the first human mAb based on transgenic mice technology (i.e., panitumumab) was only approved by the FDA and EMA in 2006 and 2007, respectively, to treat patients with colorectal cancer harboring epidermal growth factor receptor (EGFR) mutations [20].

The advent of these new technologies propelled interest in the production of recombinant Fc-fusion proteins, which are characterized by the Fc domain of the Ab conjugated with another protein such as an enzyme, cytokine or a receptor through a peptide linker, therefore increasing the stability and half-life of the bound protein and prolonging its biological effects [21]. The first Ab fusion protein, etanercept, was approved by the FDA and EMA for clinical use in patients with RA in 1998 and 2000, respectively. It comprises the Fc region of Ab conjugated with TNF-receptor 2 (TNFR2) [22]. The approval of etanercept opened up the way to the development of several recombinant protein methodologies based on the fusion of various proteins to different antibody regions, including single-chain variable fragments (scFvs), heavy-chain Abs (hcAbs), single-chain Abs (scAbs) and antigen-binding fragments (Fabs) [23].

The development of these Abs was based on the discovery of Abs with only two heavy chains (containing a single variable domain referred to as a VHH or nanobody) in the serum of camelids (alpaca, llama and camel) in 1993 [24]. Although the nanobodies have a low molecular weight (14 kDa) and bind very well to the antigen, the binding is unstable [25]. Through the development of bioengineering, the VHH from hcAbs can be isolated, maintaining the binding properties of the original hcAbs. Once isolated, a fusion of nanobodies may be generated, either by direct chemical bonding creating a 25 kDa bivalent structure or by binding to human Fc chain fragments, creating human-compatible 50 kDa structures. The bivalent nanobodies quickly proved to be very stable at high temperatures and able to bind to specific epitopes not easily accessible by conventional mAbs [26].

In 2019, the first bivalent nanobody directed against the A1 domain of the von Willebrand factor (caplacizumab) received FDA and EMA approval to treat patients with acquired thrombotic thrombocytopenic purpura (aTTP) [27,28]. Three years later, ozoralizumab was approved in Japan for patients with RA [29]. It is a trivalent bispecific nanobody that has a high binding affinity with both human TNF-α and human serum albumin [30].

Currently, different nanobody therapies are investigated in the clinical setting. It appears likely that as the breadth of nanobody targets is expanded, their utility as diagnostics or therapeutics will grow as well.

In the present review, we will discuss the historical development, as well as the present and potential future applications of all five FDA-approved biological anti-TNF-α agents (infliximab, adalimumab, etanercept, golimumab and certolizumab), which have led to major improvements for patients with RA and other autoimmune diseases, such as ankylosing spondylitis, Crohn’s disease, ulcerative colitis and psoriasis. More recently, other therapeutic areas are under evaluation, including viral infections, e.g., COVID-19, neuropsychiatric disorders and cancer [31].

The search for biomarkers for predicting anti-TNF-α responsiveness is also discussed.

## 2. Tumor Necrosis Factor-Alpha

**Figure 1:**
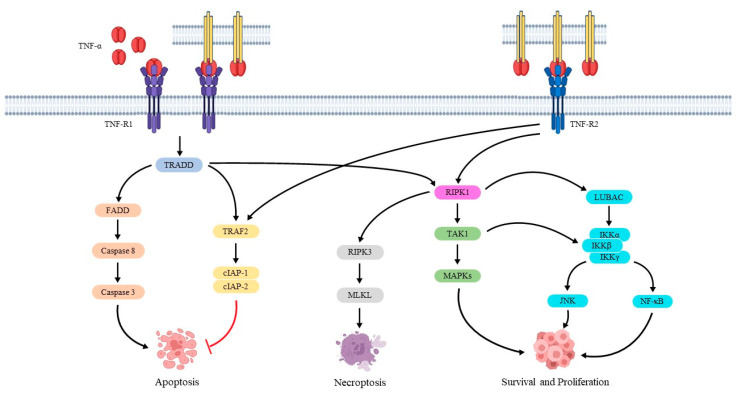
Signaling pathways of TNF-α.

The tumor necrosis factor-alpha (TNF-α) gene is located in chromosome 6. It consists of four exons, and it encodes for a pro-inflammatory and immunomodulatory transmembrane protein made up of 233 amino-acid residues [32]. TNF-α is expressed as a transmembrane precursor by macrophages but also by CD4+ T cells, mast cells, neutrophils and NK cells. When these cells are activated, TNF-α is cleaved and released by a metalloprotease enzyme known as a TNF-α-converting enzyme (TACE) to generate its soluble form of 157 amino-acid residues, which promotes inflammation. The binding of TNF-α with its receptors triggers several events, including the production of cytokines, expression of adhesion molecules, releasing of pro-coagulatory substances and production of the synthetase nitric oxide. In addition, TNF-α increases the expression of other pro-inflammatory cytokines (i.e., interleukin-1 (IL-1), IL-6 and IL-8), suppresses lipoprotein lipase in adipocytes and stimulates hepatocytes to produce acute phase proteins which sustain systemic inflammation [33,34]. The effects of TNF-α are mediated by binding to two different receptors (TNF-R1 and TNF-R2). TNF-R1 is the main mediator of TNF-α action, and it is expressed on the surface of several cell types [35]. TNF-R1 is activated by both soluble and transmembrane TNF-α forms, whereas transmembrane TNF-α activates TNF-R2 which is expressed on the surface of lymphocytes [36,37]. These receptors have similar cysteine-rich extracellular domains, although they have different intracellular domains (ICD) [38]. In the ICD of TNF-R1, there is a death domain (DD) which recruits an adaptor protein known as the TNF receptor-associated death domain (TRADD) [39]. TRADD is recognized by other adaptor proteins, including receptor-interacting protein kinase 1 (RIPK1) and TNF-R-associated factor 2 (TRAF2). These proteins make the complex I that has a key role in cell survival and proliferation. In particular, TRAF2 binds and activates the cellular inhibitor of apoptosis protein-1 (cIAP-1) and 2 (cIAP-2), which exerts a ligase activity. RIPK1 binds and actives TGFβ-activated kinase 1 (TAK1) and the linear ubiquitin chain assembly complex (LUBAC), promoting the activation of the c-Jun N-terminal kinase (JNK), mitogen-activated protein kinases (MAPKs) and nuclear factor κB (NF-κB) signaling pathways via an inhibitor of κB kinase (IKK) complex activation [40,41]. However, TRADD can also bind the Fas-associated death domain (FADD) producing complex II. This complex recruits and activates pro-caspase 8, which triggers the proteolytic cleavage of pro-caspase 3 and starts the apoptotic process [42]. In addition, the phosphorylated RIPK3 with RIPK1 can phosphorylate and activate the mixed lineage kinase domain-like protein (MLKL), inducing inflammatory and necroptosis. On the other hand, TNF-R2 lacks the DD domain. Hence, it can only promote cell survival and proliferation through the activation of MAPK, NF-κB and JNK pathways (Figure 1) [37,43].

An abnormal production of TNF-α is associated with several chronic, immunoinflammatory diseases, such as rheumatoid arthritis (RA), inflammatory bowel disease (IBD), psoriasis (PS), psoriatic arthritis (PsA) and autoimmune uveitis [44,45,46,47,48].

## 3. TNF-α Blockers

To date, five anti-TNF-α drugs have been approved: infliximab (Remicade), adalimumab (Humira), golimumab (Simponi), certolizumab pegol (Cimzia) and etanercept (Enbrel). Several studies have demonstrated the efficacy and safety profiles of these agents in different diseases. In particular, Burr and colleagues demonstrated that patients with severe ulcerative colitis treated with infliximab (10 mg/kg) showed superior endoscopic improvement compared with the placebo group (with a response rate of 0.61) [49]. Another group reported that 60% of patients with uveitis treated with adalimumab or infliximab showed a visual acuity improvement and a central macular thickness decrease of 112.70 μm [50]. Moreover, Liu and colleagues observed greater efficacy of TNF-α inhibitors than the placebo in patients with ankylosing spondylitis [51]. Finally, Fleischmann and colleagues demonstrated that the addition of TNF-α inhibitors to methotrexate in RA patients with an inadequate response to methotrexate could be associated with a greater likelihood of achieving the American College of Rheumatology 70% response criteria at 6 months compared to the addition of sulfasalazine and hydroxychloroquine [52].

Overall, these studies highlight that the use of TNF-α inhibitors is safe and well tolerated in a large percentage of patients suffering from various diseases.

### 3.1. Infliximab

**Figure 2:**
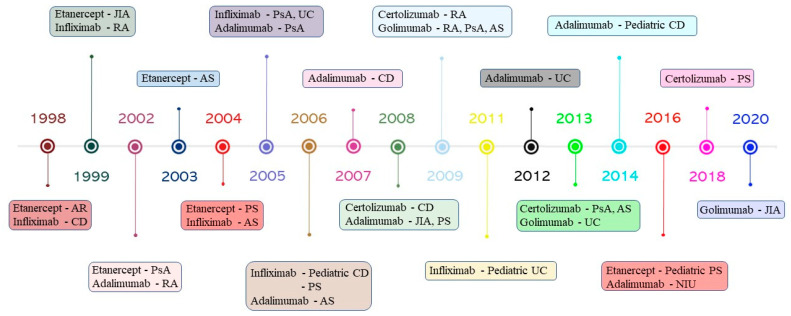
Timeline of the approval of TNF-α blockers in autoimmune diseases. AR, rheumatoid arthritis; CD, Crohn’s disease; AS, ankylosing spondylitis; PsA, psoriatic arthritis; PS, psoriasis; UC, ulcerative colitis; NIU, non-infectious uveitis; JIA, juvenile idiopathic arthritis.

Infliximab is a chimeric mAb, generated by Vilcek and Le [53], consisting of murine variable regions from the murine anti-TNF-α hybridoma A2 and human IgG1 constant regions. It binds both transmembrane and soluble TNF-α with high affinity, hindering the binding with their receptors and neutralizing their biological effects [54]. Infliximab was first approved by the FDA in 1998 for treating patients with Crohn’s disease (CD) [9]. Subsequently, it has received approval for the treatment of RA, ankylosing spondylitis (AS), psoriatic arthritis (PsA) and psoriasis (PS) patients (Figure 2) [55]. The approval was based on the results from a randomized double-blind study conducted on 108 patients, which showed that infliximab at 5 mg/kg resulted in a response rate of 81% [56]. However, infliximab administration is associated with the manifestation of severe adverse events, including pneumonia, hepatotoxicity, lymphoma and the reactivation of the tuberculosis [57].

### 3.2. Etanercept

Etanercept is a recombinant Fc-fusion protein that comprises two human TNF-R2 conjugated with the CH2 and CH3 domains of the Fc region of human IgG1, which mimics soluble TNF-R activity [58]. It was approved by the FDA for clinical practice in patients with RA in 1998 [22]. It was further approved for the treatment of juvenile idiopathic arthritis (JIA), PsA, AS and PS (Figure 2). The approval was based on the results from a randomized double-blind study conducted on 234 patients with RA. The patients were randomly assigned to receive a placebo or two doses of subcutaneous injections of etanercept (10 or 25 mg) for six months. Overall, 40% of patients treated with etanercept at 25 mg showed a significant improvement, with a 50% American College of Rheumatology (ACR) response, compared to 4% of the placebo group [59]. Etanercept is less immunogenic than infliximab; however, it produces unstable complexes due to the presence of a conformational hindrance as a result of the absence of a hinge region in its Fc region, resulting in it being weaker than other blockers [60]. In addition, it is not specific to TNF-α but can also recognize and bind other members of the lymphotoxin family, such as TNF-*β*, which is a cytokine involved in the regulation of bowel immune cells [61]. Furthermore, it should be noted that long-term administration of etanercept causes severe infections and sepsis that may lead to hospitalization or death. To prevent the risk of infections, it is administrated along with methotrexate or prednisone, but the clinical use of etanercept is not recommended for patients with active infections [62].

### 3.3. Adalimumab

In 2002, adalimumab, a fully human mAb generated using phage display technology, was approved by the FDA to treat patients with RA [63]. Subsequently, it was approved for the treatment of PsA, AS, CD, JIA, ulcerative colitis (UC) and non-infectious uveitis (NIU) (Figure 2) [64]. The efficacy and safety of adalimumab have been substantiated by the results gained from a randomized, double-blind, placebo-controlled trial. Of the 120 RA patients recruited, 89 received ascending doses of adalimumab, and 31 received a placebo. Overall, adalimumab at 10 mg/mL showed a response rate of 100%, which was significantly higher than the other groups, while no adverse events were observed compared with the placebo group [65]. Adalimumab induces a strong complement-mediated cytotoxicity and is less immunogenic that infliximab due to the absence of murine variable domains of the immunoglobulin Fab [47]. However, patients treated with adalimumab manifested several adverse events, including thrombocytopenia, leukopenia, malignancies and a reactivation of the tuberculosis, with an odds ratio of 14.6 [66].

### 3.4. Golimumab

Golimumab is a fully human mAb produced using Medarex’s UltiMab transgenic mouse platform, in which human IgG genes are inserted into the genome of engineering mice. It has a bivalent Fab region, which can bind the soluble and transmembrane form of TNF-α proteins with a higher affinity than infliximab and adalimumab, reducing both the circulating TNF-α protein levels and the binding of TNF-α with its receptors [67]. In comparison with other TNF-α blockers, it was less immunogenic than [68]. Golimumab reduces serum levels of IL-6, IL-8, serum amyloid A, serum amyloid P and ferritin and inhibits the cell surface expression of adhesion molecules, including E-selectin, intracellular adhesion molecule 1 (ICAM-1) and vascular cell adhesion molecule 1 (VCAM-1) [69]. In 2009, golimumab received FDA approval for the treatment of RA, PsA and AS [64]. More recently, it was also approved for the treatment of UC and JIA in 2013 and 2020, respectively (Figure 2). The approval was based on the results of the multicenter, randomized, double-blind, placebo-controlled GO-AFTER trial [70].

### 3.5. Certolizumab Pegol

In 2008, certolizumab pegol was approved by the FDA to treat patients with CD [71]. Its efficacy and safety were proven by the results obtained from a randomized, double-blind, placebo-controlled trial conducted on 662 patients with CD [71]. It was further approved for the treatment of RA, PsA, AS and PS (Figure 2). It is a single Fab fragment of humanized IgG1 mAb conjugated to two 20 kDa polyethylene glycol chains that increase the drug’s half-life [72]. Furthermore, it is capable of binding both soluble and transmembrane TNF-α forms, neutralizing the binding with their receptors [73]. Certolizumab pegol lacks the Fc region which may trigger the complement-mediated cytotoxicity, so it does not produce cellular lysis by inducing natural killer cell activity [74].

## 4. Adverse Events after TNF-α Blocker Use

The toxicity profile of TNF-α blockers is acceptable; however, patient surveillance continues to be warranted. Common side effects range from gastrointestinal to behavioral disturbances. Rarely, patients may also experience symptoms, such as fatigue, myalgia, nausea and anorexia which often disappear after the discontinuation of therapy [75,76,77]. Furthermore, several researchers have reported the role of anti-TNF-α agents in the development of different serious side effects, including infections, malignancies, autoimmune diseases, in particular systemic lupus erythematosus, Guillain–Barre syndrome and multiple sclerosis [78,79,80].

### 4.1. Infections

For years, researchers have been trying to understand whether patients treated with TNF-α blockers are at a greater risk of developing serious infections than the general population, reaching conflicting results. The discrepancies among the different studies could be related to several factors, such as the duration of the treatment and follow-up, the patients’ prior therapies, and the severity of the disease [81,82,83]. The respiratory system is the site mostly affected by infections in these patients. In particular, the blockade of TNF-α can cause reactivation of latent tuberculosis [84]. To reduce the risk of the reactivation of tuberculosis, current guidelines recommend screening for latent tuberculosis before starting treatment with TNF-α blockers [85]. However, none of these recommendations provide indications for choosing the correct biological treatment according to the specific risk associated with each patient [86]. Attention should also be paid to the possibility of the reactivation of the hepatitis B virus (HBV) during treatment with anti-TNF-α. The vaccination of children has proven very useful when it is administered before the onset of immunosuppressive therapy [87,88]. In this regard, in 2011, the task force of the European League Against Rheumatism (EULAR) published recommendations regarding the vaccination of adults and children with rheumatic diseases [89].

### 4.2. Demyelinating Disorders

Among the neurological manifestations associated with biological TNF inhibitors, headaches and behavioral disturbances are the most common [75]. However, a wide range of more severe neurological disorders has been associated with TNF-α inhibitors, including Guillain–Barre syndrome, peripheral neuropathies, multiple sclerosis, optic neuritis and acute transverse myelitis [90,91]. A retrospective study showed that IBD patients treated with TNF-α blockers showed a 43% increase in the incidence of multiple sclerosis versus patients who did not receive these agents [92]. The demyelination of the central and peripheral nervous system was reported more frequently after 17 months of treatment, although the causal association with anti-TNF-α therapy is still uncertain [92,93]. Indeed, the use of TNF-α inhibitors in patients with multiple sclerosis has showed a worsening of the disease [94,95,96]. To date, there are no guidelines for the management of patients who manifest this complication, and only the suspension of TNF-α inhibitors and the administration of high-dose corticosteroids are recommended [97].

### 4.3. Drug-Induced Lupus

Another adverse effect of TNF-α inhibitors is the onset of induced iatrogenic lupus (ATIL, anti-tumor necrosis factor-alpha-induced lupus). Many people who develop ATIL show variable symptoms, such as skin rashes, thrombocytopenia, leukopenia, pericarditis, pneumonia and rarely, hemolytic anemia [98,99]. These symptoms often occur in association with elevated serum levels of antinuclear antibodies and double-stranded DNA antibodies, which decrease within a few months after withdrawal of treatment. The pathogenetic mechanism by which anti-TNF-α leads to the development of ATIL is not yet clear. However, this appears to be due to the suppression of Th1 cytokine production, driving the immune response towards the production of IFN-α [100]. Furthermore, anti-TNF-α therapy may inhibit cytotoxic T cells, leading to a reduction in the elimination of autoantibody-producing B cells [101].

Although all anti-TNF-α were reported to potentially induce ATIL, several studies have shown that the onset of ATIL occurs mainly in patients taking infliximab and, in some cases, after treatment with etanercept and adalimumab [102,103,104]. In particular, the VigiBase dataset has reported that more than 30% of all ATIL cases were due to TNF-α inhibitors (12.2% infliximab, 10.7% adalimumab and 8.0% etanercept) [105]. More recently, Dai and colleagues have reported an increased ATIL incidence in CD patients treated with infliximab compared with the adalimumab group (with an incidence rate of 4.5 and 0.2%, respectively) [106].

Nowadays, it is still unclear whether TNF-α inhibitors play a role in the exacerbation of systemic lupus erythematosus. In order to ensure an accurate diagnosis, it is recommended that a thorough immunological screening is always performed before starting the treatment with TNF-α inhibitors.

### 4.4. Malignancy

The risk of malignancy associated with the use of TNF-α inhibitors is still widely debated. In fact, treatment-induced suppression of the immune system may increase the risk of cancer development. According to Bongartz and colleagues, high doses of infliximab or adalimumab increases by 3.3-fold the risk of malignancy in RA patients compared with placebo groups. However, this study did not analyze any person–year incidence rate [107]. In contrast, Burmester and colleagues showed no significant differences in cancer risk in RA patients treated with adalimumab [108].

Recently, an increased risk of cancer, especially for non-melanoma skin cancer and lymphoma, was described in patients taking anti-TNF-α agents compared with the control population [109,110]. In particular, an Icelandic case-control study suggests that RA and PS patients taking TNF-α inhibitors have an increased risk of developing in situ squamous cell carcinoma but not invasive squamous cell carcinoma or basal cell carcinoma [109]. Regarding lymphoma, an American case-control study showed that earlier use of etanercept increases the risk of non-Hodgkin lymphoma (NHL) three times [110].

Although these studies have reported an increased cancer risk in patients taking TNF-α blockers, no definitive conclusions can be drawn, as many studies have not confirmed this trend [111,112,113]. A possible explanation of these conflicting results may arise from several biases in the studies. In particular, an increased risk of lymphoma in patients with systemic inflammatory diseases taking TNF-α inhibitors could be a result of the disease process and not the effect of immunosuppressive therapy [114]. Furthermore, the increased risk of cancer could be due to other immunosuppressant drugs that are given together with TNF-α inhibitors. In fact, a higher rate of malignancies was found among IBD patients treated with the combination of TNF-α inhibitors and thiopurine (azathioprine or 6-mercaptopurine) compared to those treated without thiopurine (standardized incidence ratios of 6.0 vs. 2.5, respectively) [115]. Additionally, the patient’s family history should be investigated to identify the possible predisposition to cancer. Finally, the follow-up period should be sufficiently extended to better evaluate the effective incidence of the neoplasm. Overall, it is recommended that patients who have already had cancer take TNF-α inhibitors with caution.

In light of the enormous interest attracted by immune checkpoint inhibitors (ICI) in the treatment of cancer and the frequent occurrence of immune-related adverse events (irAEs) associated with their use, it has also been debated whether the use of biological TNF inhibitors may impact the pharmacological response to ICI and the development of irAEs. Emerging clinical and preclinical evidence suggests that at least short courses of TNF inhibitors are safe for the treatment of irAEs in patients with cancer undergoing ICI therapy. Data from preclinical studies also propose that TNF inhibition might augment the antitumor effect of ICI therapy while simultaneously ameliorating irAEs [31,116].

### 4.5. Development of Anti-Drug Antibodies (ADA)

The clinical data indicate that not all patients respond to TNF-α blocker therapy. In fact, the use of these agents is associated with a lack of clinical response in about 30–40% of naïve patients (primary therapeutic failure) [117]. It is also known that some patients responding to TNF-α blockers at the beginning of the therapy may lose sensitivity to these drugs at later phases (secondary treatment failure) [117]. The current evidence suggests that the development of anti-drug antibodies (ADA) is one of the most important factors that can account for secondary failure [118]. ADA can block the therapeutic action of the respective anti-TNF-α drug. Indeed, neutralizing ADA bind the drug to prevent it from interacting with its molecular target, while non-neutralizing ADA facilitate drug clearance, resulting in the reduction in drug concentrations below the levels needed to carry out the expected therapeutic effect [119]. Moreover, the production of ADA can lead to the development of adverse reactions. Indeed, it has been observed that the development of ADA specifically against TNF-α antagonists has been associated with acute infusion reactions, which usually occur within 24 h of TNF-α inhibitor administration [120]. Conversely, no association was highlighted among the ADA directed against anti-TNF-α drugs and delayed hypersensitivity reactions occurring 3 to 12 days after the infusion.

Maneiro and colleagues showed up to a four-fold increased risk of infusion reactions in ADA-positive patients compared with ADA-negative patients [121]. Infliximab showed the greatest immunogenic power among the currently usable TNF-α inhibitors due to the presence of murine regions in its molecular structure. Indeed, a recent meta-analysis has determined that 25% of infliximab-treated patients develop ADA [122]. Moreover, a recent study suggests that etanercept is the less immunogenic biological TNF-α inhibitor [123]. In clinical studies, the presence of anti-etanercept ADA in the serum of patients treated with this drug was found in only 1.2% of cases [122]. However, it appears that the presence of ADA has not been associated with significant reductions in the therapeutic efficacy of etanercept, even in long-term studies [124,125].

To avoid the immunogenic effects of TNF-α inhibitors, two therapeutic approaches are available: one is to switch to a second TNF-α inhibitor or agents with different mechanisms of action; another alternative strategy may be the use of immunosuppressive agents in combination with TNF-α inhibitors. In particular, several studies have shown that the co-administration of immunosuppressants (azathioprine, glucocorticoids and methotrexate) reduces immunogenicity and increases the serum levels of TNF-α inhibitors [121,122,126,127,128,129]. However, the mechanisms underlying these effects are not yet fully understood. Therefore, it would be necessary to monitor the patient and detect the presence of ADA in serum as soon as possible.

Although numerous studies have shown that the development of ADA can be associated with a loss or reduction of the therapeutic action of TNF-α inhibitors, these data should be considered with considerable caution. In fact, the presence of ADA cannot be considered a condition necessarily associated with the loss or reduction of efficacy of the respective biotechnological drug, as some patients who develop ADA against a specific TNF-α inhibitor show a good persistence of therapeutic response to treatment with the same agents [119]. The development of immune tolerance could play a key role in this mechanism. Notably, several studies have shown that one-third of patients show a transient ADA response with serum ADA levels decreasing over time [130,131,132].

Overall, the mechanisms underlying the development of ADA and their effects on therapeutic efficacy are not fully understood yet. Further studies are needed to identify possible biomarkers that could predict the development of ADA in patients after treatment with TNF-α inhibitors.

### 4.6. Other Adverse Events

Based on several clinical studies, TNF-α inhibitors seem to play a key role in the evolution and development of various pathologies. Indeed, there are post-marketing reports of the worsening of congestive heart failure (CHF) in patients treated with these agents. In particular, a randomized study suggests that infliximab increases the risk of hospitalization in patients with CHF [133]. Similar results were obtained in other clinical studies evaluating the role of etanercept [134]. Furthermore, there have been also rare cases of de novo onset of CHF in patients without pre-existing cardiovascular disease [135].

Regarding the role of TNF-α inhibitors in pregnancy, there is no evidence to suggest that treatment with these drugs, before or during pregnancy, is associated with increased risks of preterm births, congenital anomalies or poor pregnancy outcomes [136,137]. However, a prospective study revealed that treatment with TNF-α inhibitors in combination with thiopurines in pregnancy increases the risk of neonatal viral infections [138].

Finally, some adverse events may be induced by interactions between TNF-α inhibitors and other drugs. Although, most studies have observed no clinically relevant drug interactions, some evidence suggests that TNF-α inhibitors may affect cytochrome p450 (CYP450) activity [139]. In particular, the effect of TNF-α inhibitors could involve the restoration of CYP450 with direct effects on plasma concentration and the pharmacological activity of co-administered drugs which are metabolized by CYP450. Furthermore, a clinical study has demonstrated that etanercept in combination with cyclophosphamide was associated with a higher incidence of malignant solid tumors compared with standard therapy alone in patients with Wegener’s granulomatosis [140].

Drug interactions should be considered as one of the possible causes of any adverse events. When unexpected clinical responses occur, the physician should determine the serum concentrations of the drugs and adjust the dosage until the desired effect is obtained. If the dosage adjustment is ineffective, it should be replaced with one that does not interact with the other agent.

## 5. Perspectives on Future Applications of TNF-α Blockers

### 5.1. TNF-α Blockers in COVID-19 Disease

During viral infections, inflammation plays a key role in the fight against viral agents. However, TNF-α can also lead to excessive activation of immune cells and a hyperproduction of other pro-inflammatory cytokines, damaging different organs [141]. Recently, the role of TNF-α has been investigated in SARS-CoV-2 disease (COVID-19). The spike glycoproteins of SARS-CoV-2 recognise and bind to angiotensin-converting enzyme-2 (ACE-2) receptors that are expressed on the surface of alveolar, cardiac, endothelial and hematopoietic cells [142]. Then, the spike–ACE-2 complex internalizes into cell cytoplasm and allows the increase in TACE enzymatic activity [143]. Soluble TNF-α is secreted already in the early stages of the infection, underlying the “cytokine storm”, which results in hyperinflammation and tissue damage [144]. Although TNF-α plays a primary role in the onset of the cytokine cascade, anti-TNF agents are not yet clinically approved for the treatment of COVID-19 disease. However, observational studies have suggested that the administration of TNF-α blockers seems to be promising in patients with severe disease [145,146].

In particular, the Inflammatory Bowel Disease Registry (SECURE-IBD) has reported the beneficial effects of TNF-α blockers for treating patients with inflammatory bowel disease (IBD) affected by COVID-19. In particular, among 2307 IBD patients treated with anti-TNF-α monotherapy before and during COVID-19 disease and 2088 patients receiving sulfasalazine/mesalazine treatment, 8% were hospitalized and <1% died in the anti-TNF-α group, while 20% were hospitalized and 3% died in the other group [147].

In addition, the Global Rheumatology Alliance (GRA) registry has reported the beneficial effects of TNF-α blockers for treating COVID-19 patients with RA. On 12 April 2021, 1388 patients with RA received TNF-α inhibitors at the onset of COVID-19. Of these, 103 patients (7.4%) were hospitalized with ventilation, and only 36 (2.6%) died. Notably, treatment with other drugs (rituximab and Janus kinase inhibitors) has shown a higher probability of developing a severe disease than treatment with TNF-α inhibitors [148]. No associations between abatacept or interleukin 6 inhibitors and COVID-19 severity were observed [148].

Overall, these data have shown that the clinical use of TNF-α blockers is significantly associated with a lower COVID-19-related hospitalization rate compared with other drugs. However, further studies are needed to better define an immune profile of both the disease course and therapy with TNF-α antagonists. For instance, some data show that the use of TNF-α inhibitors seems to be associated with a mild humoral response to the SARS-CoV-2 vaccines and a faster lowering of the antibody titre compared with patients treated with other drugs [149,150]. Furthermore, it would be interesting to investigate the timing of the intervention to prevent the onset of the cytokine storm observed in severe COVID-19 cases.

### 5.2. Inflammation and Neuropsychiatric Disease

Several studies have recently demonstrated that a persistent inflammatory state plays a predominant role in the pathogenesis of psychiatric and neurodegenerative diseases [151,152,153,154,155]. In fact, the peripheral immune system is able to influence brain functions. This is supported by several studies which have suggested that patients with diseases associated with systemic inflammation often also have behavioral disorders [156]. Although the blood–brain barrier (BBB) selectively regulates the passage of substances to the brain, cytokines are capable of crossing it [157]. Furthermore, pro-inflammatory mediators can also be produced in the brain by resident cells, including astrocytes, microglia cells and oligodendrocytes [158]. High serum and cerebrospinal fluid levels of pro-inflammatory markers (IL-1β, IL-6, TNF-α and MIF) have been found in patients with major depression, bipolar disorder and schizophrenia [159,160,161,162,163].

Other studies have shown how TNF-α is positively associated with the severity of depressive symptoms [164]. In addition, the use of antidepressants reduces the release of circulating inflammatory factors, increasing the release of endogenous antagonists of pro-inflammatory cytokines, such as IL-10 [165,166]. Moreover, Benedetti and colleagues reported a relationship between some pro-inflammatory cytokines (TNF-α, IL-8 and IFN-γ) and white matter integrity. In particular, these molecules were significantly associated with a decrease in the integrity of the white matter (fractional anisotropy) and an increase in the permeability of the myelin sheath (radial and average diffusivity) [167].

#### TNF-α Blockers in Neuropsychiatric Disease

Based on the studies described above, several studies have recently focused on evaluating TNF-α inhibitors as potential therapeutics for behavioral disorders, obtaining encouraging results. Indeed, some studies have reported a significant reduction in anxiety and depressive-like behaviour and an improvement in cognitive function in murine models after anti-TNF-α treatment [168,169]. In line with these pre-clinical studies, similar results were obtained in numerous clinical trials [170,171]. In particular, a randomized, double-blind clinical trial revealed that patients with bipolar disorders treated with infliximab are affected by a depletion in leptin levels [172]. Moreover, the same group observed that infliximab treatment significantly reduces glutamate levels in patients with bipolar disorders [173]. Moreover, the results of these two trials suggested an increase in global cortical volume and an improvement in cognitive functions in bipolar patients after infliximab administration. However, a randomized, double-blind, placebo-controlled trial showed a significant reduction in symptom severity only in bipolar patients with a history of childhood physical abuse after 12 weeks of treatment with infliximab [174]. Furthermore, another group demonstrated that infliximab exhibits only short-lived antidepressant effects in patients with bipolarity [175]. Finally, a systematic review showed a reduction of depressive symptoms only in patients with high protein C reactive (PCR) and TNF-α levels. However, no statistically significant effects were found between the infliximab and placebo groups with low levels of inflammatory markers [176].

Although TNF-α inhibitors are unable to cross the BBB due to their molecular weight, they can bind to peripheral TNF-α, also reducing its levels and the overall activated immune cells in the brain parenchyma [177]. To bypass this hindrance, several strategies have been investigated. In particular, intracranial injections of infliximab have shown a reduction of Aβ and tau pathology and a rapid cognitive improvement in neurological dysfunction [178,179,180]. More recently, TNF-α inhibitors were fused with other mAbs acting as a BBB molecular “Trojan horse” (human insulin receptor or transferrin receptor mAbs) [181,182,183]. These mAbs undergo receptor-mediated transport across the BBB via the endogenous receptors, introducing the engineered TNF-α inhibitors into the brain [184].

Overall, although no data on the role of other TNF-α inhibitors are available, these studies highlight that the use of TNF-α as an adjunctive antidepressant therapy exhibits only partial positive results in a small group of patients with behavioral disorders. In addition, a recently study demonstrated that the use of TNF-α inhibitors could be a potential risk factor for the development of a manic episode in patients with or without psychiatric disorders [185].

### 5.3. Ongoing Trails Using TNF-α Blockers

The therapeutic use of anti-TNF-α has led to important advances for patients with several chronic inflammatory diseases. However, there are still autoimmune/immunoinflammatory diseases for which there is still no evidence of the efficacy of TNF-α inhibitors.

Numerous clinical trials are ongoing for the evaluation of TNF-α blockers in a wide range of disorders that may be characterized by an abnormal production of pro-inflammatory cytokines (Table 1). Most notably, Phase 2–3 trials are evaluating the use of TNF-α blockers in vasculitis patients suffering from Behcet’s disease, Takayasu’s arteritis, Wegener’s granulomatosis and giant cell arteritis for Pemphigus Vulgaris, as well as for the prevention of graft vs. host disease.

## 6. The Search for Biomarkers

Although TNF-α blockers are the main therapeutic option for patients with immune-mediated inflammatory diseases, they are characterized by high costs and immunosuppressive activity. Moreover, 40% of patients treated with them do not respond or show a loss of response over time [118]. For these reasons, many researchers have focused on identifying potential biomarkers that allow us to predict the patient’s response to treatment. Increased serum granulocyte–macrophage colony-stimulating factor (GM-CSF) levels have been found in 87.5% of RA patients responding to one of the TNF-α inhibitors [186]. In addition, Nguyen and colleagues showed that low pre-albumin and S100A12 levels associated with high platelet factor 4 (PF4) levels in pre-treatment could be good predictors for responses to infliximab, etanercept and adalimumab in patients with RA [187]. Another group observed a high number of myeloid-related protein 8 (MRP8) and MRP14-infiltrating macrophages in the synovium of RA patients responding to infliximab [188], whereas a meta-analysis demonstrated that an increased number of inflammatory plasma cells and macrophages, together with elevated tissue levels of the trigger receptor expressed on myeloid-1 cells (TREM-1), the chemokine type 2 receptor (CCR2) and the chemokine ligand 7 (CCL7) could be predictive of the failure of anti-TNF therapy in CD patients [189]. Furthermore, transcriptome analyses regarding patients with CD suggested a significant down-regulation of the K(lysine)-acetyltransferase 2B (KAT2B) gene, both in the tissues and peripheral blood mononuclear cells (PBMCs) in non-responders to anti-TNF therapy [190]. Recently, a systematic review regarding PsA or PS patients has reported that several single nucleotide polymorphisms (SNPs) could be predictive of a favorable response to anti-TNF-α treatment [191].

Finally, some studies have also shown that genetic variants could influence the response to TNF-α blockers. In particular, it was found that the presence of the IFNGrs2069705C allelic variant in patients with RA resulted in a better responsiveness to TNF-α inhibitors compared with patients harboring the wild-type allele [192]. Moreover, Sazonovs and colleagues revealed that the expression of the HLA-DQA1*05 variant was associated with anti-drug antibody generation in CD patients treated with infliximab and adalimumab [193]. 

Unfortunately, to date, no biomarker for anti-TNF responsiveness has been validated due to the heterogeneity of the results obtained. Hence, further studies using independent and large cohorts are needed.

## 7. Conclusions

Anti-TNF-α therapy has paved the way for a revolution in the approach to treating chronic inflammatory diseases. In fact, they represent the first biotechnological drugs used in rheumatology after the failure of conventional synthetic drugs, such as methotrexate, sulfasalazine and mesalazine. The efficacy and safety of these biological agents have been confirmed in various fields, and novel potential applications range from psychiatric and neurological disorders to viral infections, such as COVID-19. Novel TNF-α blockers are also currently under development, including AVX-470, L19TNFa, Ozoralizumab, 99mTc and XPro1595. Despite considerable progress, there is still an area of unmet need, for instance, cases in which the therapeutic response is insufficient or in which there are specific clinical conditions of patients or the need to control specific aspects of the disease. This explains the wide range of drugs available and the attention of the reference guidelines to the specificities of the patient, focusing on the increasingly tailored therapy. Due to this, a great effort is being made in the identification of biomarkers to be used as prognostic tools in patients eligible for treatment with TNF-α inhibitors. Secondly, biotechnological therapy is burdened by the high direct costs of the drug. The lack of patent protection for the first TNF-α inhibitors has allowed the introduction of economically affordable biosimilars, such as ABP 501 (Amjevita- Amgen Inc., Thousand Oaks, CA, USA), ABP 710 (Avsola- Amgen Inc., Thousand Oaks, CA, USA), BI 695501 (Cyltezo- Boehringer Ingelheim International GmbH, Ingelheim am Rhein, Germany), CHS-1420 (Yusimri- Coherus BioSciences Inc., Redwood City, CA, USA), CT-P13 (Inflectra- Celltrion Healthcare, Lake Forest, IL, USA), FKB327 (Hulio- Mylan Inc., Canonsburg, PA, USA), GP2017 (Hyrimoz- Sandoz, Basilea, Switzerland), PF-06410293 (Abrilada- Pfizer Inc., New York, NY, USA), PF-06438179 (Zessly- Sandoz GmbH, Kundl, Austria), SB2 (Flixabi- Samsung Bioepis, Incheon, Republic of Korea), SB4 (Brenzys- Samsung Bioepis, Incheon, Republic of Korea) and SB5 (Imraldi- Biogen-Samsung Bioepis, Incheon, Republic of Korea). The availability of biosimilar agents has allowed a drastic and important reduction in pharmacological expenditure, making TNF-α blockers widely available and accessible to the population. To date, they are an integral part of the biological therapies available. However, although biosimilars are approved on the basis of similar mechanisms of action, efficacy and safety with their reference products, many clinicians remain hesitant to suggest biosimilars as a viable treatment option due to the nocebo effect that could occur in patients following the switch to biosimilars. For this reason, it is necessary that clinicians have full and complete knowledge of the scientific principles that support these agents, their clinical development, approval and safety monitoring.

## Figures and Tables

**Table 1:** Ongoing clinical studies on future applications of TNF-α blockers registered at https://clinicaltrials.gov (accessed on 2 January 2023).

NCT Number	Interventions	Conditions	Phases
NCT03371095	Infliximab	Behcet’s Disease and Vasculitis	Phase 3
NCT03180957	Adalimumab	Dupuytren’s Disease	Phase 2
NCT02457585	Infliximab	Takayasu’s Arteritis	Phase 2
NCT01730495	Etanercept	Chronic Fatigue Syndrome and Myalgic Encephalomyelitis	Phase 2
NCT01423591	Infliximab	Polymyalgia Rheumatica	Phase 3
NCT00753103	Infliximab	Wegener’s Granulomatosis, Renal Limited Vasculitis and Microscopic Polyangiitis	Phase 2
NCT00726375	Etanercept	Acute Graft vs. Host Disease	Phase 3
NCT00604864	Infliximab	Endometriosis	Phase 2
NCT00368264	Infliximab	Lupus Erythematosus Systemic and Lupus Nephritis	Phase 2/Phase 3
NCT00329823	Etanercept	Hidradenitis Suppurativa	Phase 2
NCT00305539	Adalimumab	Giant Cell Arteritis	Phase 3
NCT00228839	Infliximab	Graft vs. Host Disease	Phase 1
NCT00203359	Etanercept	Alzheimer’s Disease	Phase 1
NCT00203320	Etanercept	Alzheimer’s Disease	Phase 1
NCT00135720	Etanercept	Pemphigus Vulgaris	Phase 2
NCT00031551	Etanercept	Stomatitis	Phase 2

## Data Availability

Not applicable.

## References

[1] Köhler G., Milstein C. (1975). Continuous Cultures of Fused Cells Secreting Antibody of Predefined Specificity. Nature.

[2] Ecker D.M., Jones S.D., Levine H.L. (2015). The Therapeutic Monoclonal Antibody Market. MAbs.

[3] Sharma S.K., Bagshawe K.D., Melton R.G., Sherwood R.F. (1992). Human Immune Response to Monoclonal Antibody-Enzyme Conjugates in ADEPT Pilot Clinical Trial. Cell Biophys..

[4] Boulianne G.L., Hozumi N., Shulman M.J. (1984). Production of Functional Chimaeric Mouse/Human Antibody. Nature.

[5] Foster R.H., Wiseman L.R. (1998). Abciximab. Drugs.

[6] James J.S., Dubs G. (1997). FDA Approves New Kind of Lymphoma Treatment. Food and Drug Administration. AIDS Treat. News.

[7] Sharma V., Deore V.D., Deore S.V., Martin I.G. (2020). The New Drug Lag: EU Lags in Review Times of Monoclonal Antibodies. Ther. Innov. Regul. Sci..

[8] Salles G., Barrett M., Foà R., Maurer J., O’Brien S., Valente N., Wenger M., Maloney D.G. (2017). Rituximab in B-Cell Hematologic Malignancies: A Review of 20 Years of Clinical Experience. Adv. Ther..

[9] Kornbluth A. (1998). Infliximab Approved for Use in Crohnʼs Disease: A Report on the FDA GI Advisory Committee Conference. Inflamm. Bowel Dis..

[10] Jones P.T., Dear P.H., Foote J., Neuberger M.S., Winter G. (1986). Replacing the Complementarity-Determining Regions in a Human Antibody with Those from a Mouse. Nature.

[11] Hwang W.Y.K., Foote J. (2005). Immunogenicity of Engineered Antibodies. Methods.

[12] Kuramochi T., Igawa T., Tsunoda H., Hattori K. (2019). Humanization and Simultaneous Optimization of Monoclonal Antibody. Human Monoclonal Antibodies: Methods and Protocols.

[13] (1998). Monoclonal Antibody Approved for Renal Transplants. Am. J. Health Pharm..

[14] Li J., Zhu Z. (2010). Research and Development of next Generation of Antibody-Based Therapeutics. Acta Pharmacol. Sin..

[15] Zhang W., Feng J., Li Y., Guo N., Shen B. (2005). Humanization of an Anti-Human TNF-α Antibody by Variable Region Resurfacing with the Aid of Molecular Modeling. Mol. Immunol..

[16] Lonberg N., Taylor L.D., Harding F.A., Trounstine M., Higgins K.M., Schramm S.R., Kuo C.-C., Mashayekh R., Wymore K., McCabe J.G. (1994). Antigen-Specific Human Antibodies from Mice Comprising Four Distinct Genetic Modifications. Nature.

[17] Chao G., Cochran J.R., Dane Wittrup K. (2004). Fine Epitope Mapping of Anti-Epidermal Growth Factor Receptor Antibodies Through Random Mutagenesis and Yeast Surface Display. J. Mol. Biol..

[18] Green L.L., Hardy M.C., Maynard-Currie C.E., Tsuda H., Louie D.M., Mendez M.J., Abderrahim H., Noguchi M., Smith D.H., Zeng Y. (1994). Antigen–Specific Human Monoclonal Antibodies from Mice Engineered with Human Ig Heavy and Light Chain YACs. Nat. Genet..

[19] Lonberg N. (2005). Human Antibodies from Transgenic Animals. Nat. Biotechnol..

[20] Giusti R.M., Shastri K.A., Cohen M.H., Keegan P., Pazdur R. (2007). FDA Drug Approval Summary: Panitumumab (Vectibix^TM^). Oncologist.

[21] Jafari R., Zolbanin N.M., Rafatpanah H., Majidi J., Kazemi T. (2017). Fc-Fusion Proteins in Therapy: An Updated View. Curr. Med. Chem..

[22] Zhao S., Mysler E., Moots R.J. (2018). Etanercept for the Treatment of Rheumatoid Arthritis. Immunotherapy.

[23] Goulet D.R., Atkins W.M. (2020). Considerations for the Design of Antibody-Based Therapeutics. J. Pharm. Sci..

[24] Hamers-Casterman C., Atarhouch T., Muyldermans S., Robinson G., Hammers C., Songa E.B., Bendahman N., Hammers R. (1993). Naturally Occurring Antibodies Devoid of Light Chains. Nature.

[25] Muyldermans S. (2013). Nanobodies: Natural Single-Domain Antibodies. Annu. Rev. Biochem..

[26] Arbabi-Ghahroudi M. (2022). Camelid Single-Domain Antibodies: Promises and Challenges as Lifesaving Treatments. Int. J. Mol. Sci..

[27] Morrison C. (2019). Nanobody Approval Gives Domain Antibodies a Boost. Nat. Rev. Drug Discov..

[28] Jovčevska I., Muyldermans S. (2020). The Therapeutic Potential of Nanobodies. BioDrugs.

[29] Keam S.J. (2023). Ozoralizumab: First Approval. Drugs.

[30] Ishiwatari-Ogata C., Kyuuma M., Ogata H., Yamakawa M., Iwata K., Ochi M., Hori M., Miyata N., Fujii Y. (2022). Ozoralizumab, a Humanized Anti-TNFα NANOBODY® Compound, Exhibits Efficacy Not Only at the Onset of Arthritis in a Human TNF Transgenic Mouse but Also During Secondary Failure of Administration of an Anti-TNFα IgG. Front. Immunol..

[31] Chen A.Y., Wolchok J.D., Bass A.R. (2021). TNF in the Era of Immune Checkpoint Inhibitors: Friend or Foe?. Nat. Rev. Rheumatol..

[32] Pennica D., Nedwin G.E., Hayflick J.S., Seeburg P.H., Derynck R., Palladino M.A., Kohr W.J., Aggarwal B.B., Goeddel D.V. (1984). Human Tumour Necrosis Factor: Precursor Structure, Expression and Homology to Lymphotoxin. Nature.

[33] Tang B.-Y., Ge J., Wu Y., Wen J., Tang X.-H. (2022). The Role of ADAM17 in Inflammation-Related Atherosclerosis. J. Cardiovasc. Transl. Res..

[34] Bradley J. (2008). TNF-Mediated Inflammatory Disease. J. Pathol..

[35] Ye L.-L., Wei X.-S., Zhang M., Niu Y.-R., Zhou Q. (2018). The Significance of Tumor Necrosis Factor Receptor Type II in CD8+ Regulatory T Cells and CD8+ Effector T Cells. Front. Immunol..

[36] Ticha O., Slanina P., Moos L., Stichova J., Vlkova M., Bekeredjian-Ding I. (2021). TNFR2 Expression Is a Hallmark of Human Memory B Cells with Suppressive Function. Eur. J. Immunol..

[37] He T., Zhao Y., Zhao P., Zhao L., Zakaria J., Wang K. (2022). Signaling Pathway(s) of TNFR2 Required for the Immunoregulatory Effect of CD4+Foxp3+ Regulatory T Cells. Int. Immunopharmacol..

[38] Varfolomeev E., Vucic D. (2018). Intracellular Regulation of TNF Activity in Health and Disease. Cytokine.

[39] Hsu H., Xiong J., Goeddel D.V. (1995). The TNF Receptor 1-Associated Protein TRADD Signals Cell Death and NF-ΚB Activation. Cell.

[40] Park Y.C., Ye H., Hsia C., Segal D., Rich R.L., Liou H.-C., Myszka D.G., Wu H. (2000). A Novel Mechanism of TRAF Signaling Revealed by Structural and Functional Analyses of the TRADD–TRAF2 Interaction. Cell.

[41] Jackson-Bernitsas D.G., Ichikawa H., Takada Y., Myers J.N., Lin X.L., Darnay B.G., Chaturvedi M.M., Aggarwal B.B. (2007). Evidence That TNF-TNFR1-TRADD-TRAF2-RIP-TAK1-IKK Pathway Mediates Constitutive NF-ΚB Activation and Proliferation in Human Head and Neck Squamous Cell Carcinoma. Oncogene.

[42] Bender L.M., Morgan M.J., Thomas L.R., Liu Z.-G., Thorburn A. (2005). The Adaptor Protein TRADD Activates Distinct Mechanisms of Apoptosis from the Nucleus and the Cytoplasm. Cell Death Differ..

[43] Dondelinger Y., Aguileta M.A., Goossens V., Dubuisson C., Grootjans S., Dejardin E., Vandenabeele P., Bertrand M.J.M. (2013). RIPK3 Contributes to TNFR1-Mediated RIPK1 Kinase-Dependent Apoptosis in Conditions of CIAP1/2 Depletion or TAK1 Kinase Inhibition. Cell Death Differ..

[44] Choy E.H.S., Panayi G.S. (2001). Cytokine Pathways and Joint Inflammation in Rheumatoid Arthritis. N. Engl. J. Med..

[45] Adegbola S.O., Sahnan K., Warusavitarne J., Hart A., Tozer P. (2018). Anti-TNF Therapy in Crohn’s Disease. Int. J. Mol. Sci..

[46] Sands B.E., Kaplan G.G. (2007). The Role of TNFα in Ulcerative Colitis. J. Clin. Pharmacol..

[47] Levin A.D., Wildenberg M.E., van den Brink G.R. (2016). Mechanism of Action of Anti-TNF Therapy in Inflammatory Bowel Disease. J. Crohn’s Colitis.

[48] Celis R., Cuervo A., Ramírez J., Cañete J.D. (2019). Psoriatic Synovitis: Singularity and Potential Clinical Implications. Front. Med..

[49] Burr N.E., Gracie D.J., Black C.J., Ford A.C. (2022). Efficacy of Biological Therapies and Small Molecules in Moderate to Severe Ulcerative Colitis: Systematic Review and Network Meta-Analysis. Gut.

[50] Hu Y., Huang Z., Yang S., Chen X., Su W., Liang D. (2020). Effectiveness and Safety of Anti-Tumor Necrosis Factor-Alpha Agents Treatment in Behcets’ Disease-Associated Uveitis: A Systematic Review and Meta-Analysis. Front. Pharmacol..

[51] Liu W., Wu Y., Zhang L., Liu X., Xue B., Liu B., Wang Y., Ji Y. (2016). Efficacy and Safety of TNF-α Inhibitors for Active Ankylosing Spondylitis Patients: Multiple Treatment Comparisons in a Network Meta-Analysis. Sci. Rep..

[52] Fleischmann R., Tongbram V., van Vollenhoven R., Tang D.H., Chung J., Collier D., Urs S., Ndirangu K., Wells G., Pope J. (2017). Systematic Review and Network Meta-Analysis of the Efficacy and Safety of Tumour Necrosis Factor Inhibitor–Methotrexate Combination Therapy versus Triple Therapy in Rheumatoid Arthritis. RMD Open.

[53] Malaviya A., Mehra N. (2018). A Fascinating Story of the Discovery & Development of Biologicals for Use in Clinical Medicine. Indian J. Med. Res..

[54] Zidi I., Mestiri S., Bartegi A., Amor N. (2010). Ben TNF-α and Its Inhibitors in Cancer. Med. Oncol..

[55] St. Clair E.W., van der Heijde D.M.F.M., Smolen J.S., Maini R.N., Bathon J.M., Emery P., Keystone E., Schiff M., Kalden J.R., Wang B. (2004). Combination of Infliximab and Methotrexate Therapy for Early Rheumatoid Arthritis: A Randomized, Controlled Trial. Arthritis Rheum..

[56] Targan S.R., Hanauer S.B., van Deventer S.J.H., Mayer L., Present D.H., Braakman T., DeWoody K.L., Schaible T.F., Rutgeerts P.J. (1997). A Short-Term Study of Chimeric Monoclonal Antibody CA2 to Tumor Necrosis Factor α for Crohn’s Disease. N. Engl. J. Med..

[57] Bratcher J.M., Korelitz B.I. (2006). Toxicity of Infliximab in the Course of Treatment of Crohn’s Disease. Expert Opin. Drug Saf..

[58] Goffe B., Cather J.C. (2003). Etanercept: An Overview. J. Am. Acad. Dermatol..

[59] Moreland L.W. (1999). Etanercept Therapy in Rheumatoid Arthritis. Ann. Intern. Med..

[60] Horiuchi T., Mitoma H., Harashima S.-i., Tsukamoto H., Shimoda T. (2010). Transmembrane TNF-: Structure, Function and Interaction with Anti-TNF Agents. Rheumatology.

[61] Koroleva E.P., Fu Y.-X., Tumanov A.V. (2018). Lymphotoxin in Physiology of Lymphoid Tissues—Implication for Antiviral Defense. Cytokine.

[62] Goffe B. (2004). Etanercept (Enbrel)—An Update. Skin Ther. Lett..

[63] (2003). Recombinant DNA Product for Rheumatoid Arthritis.

[64] Nelson A.L., Dhimolea E., Reichert J.M. (2010). Development Trends for Human Monoclonal Antibody Therapeutics. Nat. Rev. Drug Discov..

[65] den Broeder A., van de Putte L., Rau R., Schattenkirchner M., Van Riel P., Sander O., Binder C., Fenner H., Bankmann Y., Velagapudi R. (2002). A Single Dose, Placebo Controlled Study of the Fully Human Anti-Tumor Necrosis Factor-Alpha Antibody Adalimumab (D2E7) in Patients with Rheumatoid Arthritis. J. Rheumatol..

[66] Wallis R.S. (2008). Tumour Necrosis Factor Antagonists: Structure, Function, and Tuberculosis Risks. Lancet Infect. Dis..

[67] Xu Z., Vu T., Lee H., Hu C., Ling J., Yan H., Baker D., Beutler A., Pendley C., Wagner C. (2009). Population Pharmacokinetics of Golimumab, an Anti-Tumor Necrosis Factor-α Human Monoclonal Antibody, in Patients With Psoriatic Arthritis. J. Clin. Pharmacol..

[68] Shealy D.J., Cai A., Staquet K., Baker A., Lacy E.R., Johns L., Vafa O., Gunn G., Tam S., Sague S. (2010). Characterization of Golimumab, a Human Monoclonal Antibody Specific for Human Tumor Necrosis Factor α. MAbs.

[69] Oldfield V., Plosker G.L. (2009). Golimumab. BioDrugs.

[70] Smolen J.S., Kay J., Doyle M.K., Landewé R., Matteson E.L., Wollenhaupt J., Gaylis N., Murphy F.T., Neal J.S., Zhou Y. (2009). Golimumab in Patients with Active Rheumatoid Arthritis after Treatment with Tumour Necrosis Factor α Inhibitors (GO-AFTER Study): A Multicentre, Randomised, Double-Blind, Placebo-Controlled, Phase III Trial. Lancet.

[71] Lang L. (2008). FDA Approves Cimzia to Treat Crohn’s Disease. Gastroenterology.

[72] Palframan R., Airey M., Moore A., Vugler A., Nesbitt A. (2009). Use of Biofluorescence Imaging to Compare the Distribution of Certolizumab Pegol, Adalimumab, and Infliximab in the Inflamed Paws of Mice with Collagen-Induced Arthritis. J. Immunol. Methods.

[73] Nesbitt A., Fossati G., Bergin M., Stephens P., Stephens S., Foulkes R., Brown D., Robinson M., Bourne T. (2007). Mechanism of Action of Certolizumab Pegol (CDP870): In Vitro Comparison with Other Anti-Tumor Necrosis Factor α Agents. Inflamm. Bowel Dis..

[74] Lis K., Kuzawińska O., Bałkowiec-Iskra E. (2014). State of the Art Paper Tumor Necrosis Factor Inhibitors – State of Knowledge. Arch. Med. Sci..

[75] Verazza S., Davì S., Consolaro A., Bovis F., Insalaco A., Magni-Manzoni S., Nicolai R., Marafon D.P., De Benedetti F., Gerloni V. (2016). Disease Status, Reasons for Discontinuation and Adverse Events in 1038 Italian Children with Juvenile Idiopathic Arthritis Treated with Etanercept. Pediatr. Rheumatol..

[76] Pastore S., Naviglio S., Canuto A., Lepore L., Martelossi S., Ventura A., Taddio A. (2018). Serious Adverse Events Associated with Anti-Tumor Necrosis Factor Alpha Agents in Pediatric-Onset Inflammatory Bowel Disease and Juvenile Idiopathic Arthritis in A Real-Life Setting. Pediatr. Drugs.

[77] Foeldvari I., Constantin T., Vojinović J., Horneff G., Chasnyk V., Dehoorne J., Panaviene V., Sušić G., Stanevicha V., Kobusinska K. (2019). Etanercept Treatment for Extended Oligoarticular Juvenile Idiopathic Arthritis, Enthesitis-Related Arthritis, or Psoriatic Arthritis: 6-Year Efficacy and Safety Data from an Open-Label Trial. Arthritis Res. Ther..

[78] Kaltsonoudis E., Pelechas E., Voulgari P.V., Drosos A.A. (2022). Neuroinflammatory Events after Anti-TNFα Therapy. Ann. Rheum. Dis..

[79] Bonovas S., Minozzi S., Lytras T., González-Lorenzo M., Pecoraro V., Colombo S., Polloni I., Moja L., Cinquini M., Marino V. (2016). Risk of Malignancies Using Anti-TNF Agents in Rheumatoid Arthritis, Psoriatic Arthritis, and Ankylosing Spondylitis: A Systematic Review and Meta-Analysis. Expert Opin. Drug Saf..

[80] Li J., Zhang Z., Wu X., Zhou J., Meng D., Zhu P. (2021). Risk of Adverse Events After Anti-TNF Treatment for Inflammatory Rheumatological Disease. A Meta-Analysis. Front. Pharmacol..

[81] Doran M.F., Crowson C.S., Pond G.R., O’Fallon W.M., Gabriel S.E. (2002). Frequency of Infection in Patients with Rheumatoid Arthritis Compared with Controls: A Population-Based Study. Arthritis Rheum..

[82] Becker I., Horneff G. (2017). Risk of Serious Infection in Juvenile Idiopathic Arthritis Patients Associated With Tumor Necrosis Factor Inhibitors and Disease Activity in the German Biologics in Pediatric Rheumatology Registry. Arthritis Care Res..

[83] Lee W.-J., Lee T.A., Suda K.J., Calip G.S., Briars L., Schumock G.T. (2018). Risk of Serious Bacterial Infection Associated with Tumour Necrosis Factor-Alpha Inhibitors in Children with Juvenile Idiopathic Arthritis. Rheumatology.

[84] Calzada-Hernández J., Anton-López J., Bou-Torrent R., Iglesias-Jiménez E., Ricart-Campos S., Martín de Carpi J., Torrente-Segarra V., Sánchez-Manubens J., Giménez-Roca C., Rozas-Quesada L. (2015). Tuberculosis in Pediatric Patients Treated with Anti-TNFα Drugs: A Cohort Study. Pediatr. Rheumatol..

[85] Iannone F., Cantini F., Lapadula G. (2014). Diagnosis of Latent Tuberculosis and Prevention of Reactivation in Rheumatic Patients Receiving Biologic Therapy: International Recommendations. J. Rheumatol. Suppl..

[86] Singh J.A., Furst D.E., Bharat A., Curtis J.R., Kavanaugh A.F., Kremer J.M., Moreland L.W., O’Dell J., Winthrop K.L., Beukelman T. (2012). 2012 Update of the 2008 American College of Rheumatology Recommendations for the Use of Disease-Modifying Antirheumatic Drugs and Biologic Agents in the Treatment of Rheumatoid Arthritis. Arthritis Care Res..

[87] Lee Y.H., Bae S.-C., Song G.G. (2013). Hepatitis B Virus Reactivation in HBsAg-Positive Patients with Rheumatic Diseases Undergoing Anti-Tumor Necrosis Factor Therapy or DMARDs. Int. J. Rheum. Dis..

[88] Shah R., Ho E.Y., Kramer J.R., Richardson P., Sansgiry S., El-Serag H.B., Hou J.K. (2018). Hepatitis B Virus Screening and Reactivation in a National VA Cohort of Patients with Inflammatory Bowel Disease Treated with Tumor Necrosis Factor Antagonists. Dig. Dis. Sci..

[89] Heijstek M.W., Ott de Bruin L.M., Bijl M., Borrow R., van der Klis F., Koné-Paut I., Fasth A., Minden K., Ravelli A., Abinun M. (2011). EULAR Recommendations for Vaccination in Paediatric Patients with Rheumatic Diseases. Ann. Rheum. Dis..

[90] Solomon A.J., Spain R.I., Kruer M.C., Bourdette D. (2011). Inflammatory Neurological Disease in Patients Treated with Tumor Necrosis Factor Alpha Inhibitors. Mult. Scler. J..

[91] Piusińska-Macoch R. (2013). Neurological Complications during Treatment of the Tumor Necrosis Alpha Inhibitors. Pol. Merkur. Lekarski.

[92] Avasarala J., Guduru Z., McLouth C.J., Wilburn A., Talbert J., Sutton P., Sokola B.S. (2021). Use of Anti-TNF-α Therapy in Crohn’s Disease Is Associated with Increased Incidence of Multiple Sclerosis. Mult. Scler. Relat. Disord..

[93] Kunchok A., Aksamit A.J., Davis J.M., Kantarci O.H., Keegan B.M., Pittock S.J., Weinshenker B.G., McKeon A. (2020). Association Between Tumor Necrosis Factor Inhibitor Exposure and Inflammatory Central Nervous System Events. JAMA Neurol..

[94] Zahid M., Busmail A., Penumetcha S.S., Ahluwalia S., Irfan R., Khan S.A., Rohit Reddy S., Vasquez Lopez M.E., Mohammed L. (2021). Tumor Necrosis Factor Alpha Blockade and Multiple Sclerosis: Exploring New Avenues. Cureus.

[95] Arnett H.A., Mason J., Marino M., Suzuki K., Matsushima G.K., Ting J.P.-Y. (2001). TNFα Promotes Proliferation of Oligodendrocyte Progenitors and Remyelination. Nat. Neurosci..

[96] van Oosten B.W., Barkhof F., Truyen L., Boringa J.B., Bertelsmann F.W., von Blomberg B.M.E., Woody J.N., Hartung H.-P., Polman C.H. (1996). Increased MRI Activity and Immune Activation in Two Multiple Sclerosis Patients Treated with the Monoclonal Anti-Tumor Necrosis Factor Antibody CA2. Neurology.

[97] Kunzmann S., Warmuth-Metz M., Girschick H.J. (2005). Cerebral Demyelination in Association with TNF-inhibition Therapy in a 5-year-old Girl with Aseptic Meningitis as the First Symptom of Still’s Disease. Scand. J. Rheumatol..

[98] Costa M.F., Said N.R., Zimmermann B. (2008). Drug-Induced Lupus Due to Anti-Tumor Necrosis Factor α Agents. Semin. Arthritis Rheum..

[99] Solhjoo M., Goyal A., Chauhan K. (2022). Drug-Induced Lupus Erythematosus.

[100] Williams E.L., Gadola S., Edwards C.J. (2009). Anti-TNF-Induced Lupus. Rheumatology.

[101] Via C.S., Shustov A., Rus V., Lang T., Nguyen P., Finkelman F.D. (2001). In Vivo Neutralization of TNF-α Promotes Humoral Autoimmunity by Preventing the Induction of CTL. J. Immunol..

[102] Saka Y., Taniguchi Y., Nagahara Y., Yamashita R., Karasawa M., Naruse T., Watanabe Y. (2017). Rapidly Progressive Lupus Nephritis Associated with Golimumab in a Patient with Systemic Lupus Erythematosus and Rheumatoid Arthritis. Lupus.

[103] Bykerk V.P., Cush J., Winthrop K., Calabrese L., Lortholary O., de Longueville M., van Vollenhoven R., Mariette X. (2015). Update on the Safety Profile of Certolizumab Pegol in Rheumatoid Arthritis: An Integrated Analysis from Clinical Trials. Ann. Rheum. Dis..

[104] Shovman O., Tamar S., Amital H., Watad A., Shoenfeld Y. (2018). Diverse Patterns of Anti-TNF-α-Induced Lupus: Case Series and Review of the Literature. Clin. Rheumatol..

[105] Arnaud L., Mertz P., Gavand P.-E., Martin T., Chasset F., Tebacher-Alt M., Lambert A., Muller C., Sibilia J., Lebrun-Vignes B. (2019). Drug-Induced Systemic Lupus: Revisiting the Ever-Changing Spectrum of the Disease Using the WHO Pharmacovigilance Database. Ann. Rheum. Dis..

[106] Dai C., Wang Y., Tian W., Huang Y.-H., Jiang M. (2022). The Incidence, Clinical Characteristics and Serological Characteristics of Anti-Tumor Necrosis Factor-Induced Lupus in Patients with Inflammatory Bowel Disease: A Systematic Review and Meta-Analysis. Int. Immunopharmacol..

[107] Bongartz T., Sutton A.J., Sweeting M.J., Buchan I., Matteson E.L., Montori V. (2006). Anti-TNF Antibody Therapy in Rheumatoid Arthritis and the Risk of Serious Infections and Malignancies. JAMA.

[108] Burmester G.R., Mease P., Dijkmans B.A.C., Gordon K., Lovell D., Panaccione R., Perez J., Pangan A.L. (2009). Adalimumab Safety and Mortality Rates from Global Clinical Trials of Six Immune-Mediated Inflammatory Diseases. Ann. Rheum. Dis..

[109] Adalsteinsson J.A., Muzumdar S., Waldman R., Hu C., Wu R., Ratner D., Ungar J., Silverberg J.I., Olafsdottir G.H., Kristjansson A.K. (2021). Anti–Tumor Necrosis Factor Therapy Is Associated with Increased in Situ Squamous Cell Carcinoma of the Skin: A Population-Based Case-Control Study. J. Am. Acad. Dermatol..

[110] Calip G.S., Patel P.R., Adimadhyam S., Xing S., Wu Z., Sweiss K., Schumock G.T., Lee T.A., Chiu B.C.-H. (2018). Tumor Necrosis Factor-Alpha Inhibitors and Risk of Non-Hodgkin Lymphoma in a Cohort of Adults with Rheumatologic Conditions. Int. J. Cancer.

[111] Muller M., D’Amico F., Bonovas S., Danese S., Peyrin-Biroulet L. (2021). TNF Inhibitors and Risk of Malignancy in Patients with Inflammatory Bowel Diseases: A Systematic Review. J. Crohn’s Colitis.

[112] Choi B., Park H.J., Song Y.-K., Oh Y.-J., Kim I.-W., Oh J.M. (2022). The Risk of Newly Diagnosed Cancer in Patients with Rheumatoid Arthritis by TNF Inhibitor Use: A Nationwide Cohort Study. Arthritis Res. Ther..

[113] Mercer L.K., Lunt M., Low A.L.S., Dixon W.G., Watson K.D., Symmons D.P.M., Hyrich K.L. (2015). Risk of Solid Cancer in Patients Exposed to Anti-Tumour Necrosis Factor Therapy: Results from the British Society for Rheumatology Biologics Register for Rheumatoid Arthritis. Ann. Rheum. Dis..

[114] Franks A.L., Slansky J.E. (2012). Multiple Associations between a Broad Spectrum of Autoimmune Diseases, Chronic Inflammatory Diseases and Cancer. Anticancer Res..

[115] Beukelman T., Xie F., Chen L., Horton D.B., Lewis J.D., Mamtani R., Mannion M.M., Saag K.G., Curtis J.R. (2018). Risk of Malignancy Associated with Paediatric Use of Tumour Necrosis Factor Inhibitors. Ann. Rheum. Dis..

[116] Draghi A., Borch T.H., Radic H.D., Chamberlain C.A., Gokuldass A., Svane I.M., Donia M. (2019). Differential Effects of Corticosteroids and Anti-TNF on Tumor-specific Immune Responses: Implications for the Management of IrAEs. Int. J. Cancer.

[117] Wong U., Cross R.K. (2017). Primary and Secondary Nonresponse to Infliximab: Mechanisms and Countermeasures. Expert Opin. Drug Metab. Toxicol..

[118] Fine S., Papamichael K., Cheifetz A.S. (2019). Etiology and Management of Lack or Loss of Response to Anti-Tumor Necrosis Factor Therapy in Patients With Inflammatory Bowel Disease. Gastroenterol. Hepatol..

[119] Atiqi S., Hooijberg F., Loeff F.C., Rispens T., Wolbink G.J. (2020). Immunogenicity of TNF-Inhibitors. Front. Immunol..

[120] Jani M., Dixon W.G., Chinoy H. (2018). Drug Safety and Immunogenicity of Tumour Necrosis Factor Inhibitors: The Story so Far. Rheumatology.

[121] Maneiro J.R., Salgado E., Gomez-Reino J.J. (2013). Immunogenicity of Monoclonal Antibodies Against Tumor Necrosis Factor Used in Chronic Immune-Mediated Inflammatory Conditions. JAMA Intern. Med..

[122] Thomas S.S., Borazan N., Barroso N., Duan L., Taroumian S., Kretzmann B., Bardales R., Elashoff D., Vangala S., Furst D.E. (2015). Comparative Immunogenicity of TNF Inhibitors: Impact on Clinical Efficacy and Tolerability in the Management of Autoimmune Diseases. A Systematic Review and Meta-Analysis. BioDrugs.

[123] Bodio C., Grossi C., Pregnolato F., Favalli E.G., Biggioggero M., Marchesoni A., Murgo A., Filippini M., Migliorini P., Caporali R. (2020). Personalized Medicine in Rheumatoid Arthritis: How Immunogenicity Impacts Use of TNF Inhibitors. Autoimmun. Rev..

[124] Vincent F.B., Morand E.F., Murphy K., Mackay F., Mariette X., Marcelli C. (2013). Antidrug Antibodies (ADAb) to Tumour Necrosis Factor (TNF)-Specific Neutralising Agents in Chronic Inflammatory Diseases: A Real Issue, a Clinical Perspective. Ann. Rheum. Dis..

[125] Klareskog L., Gaubitz M., Rodríguez-Valverde V., Malaise M., Dougados M., Wajdula J. (2011). Etanercept Study 301 Investigators Assessment of Long-Term Safety and Efficacy of Etanercept in a 5-Year Extension Study in Patients with Rheumatoid Arthritis. Clin. Exp. Rheumatol..

[126] Berkhout L.C., L’Ami M.J., Wolbink G.J., Rispens T. (2021). Comment on ‘Sustained Discontinuation of Infliximab with a Raising-Dose Strategy after Obtaining Remission in Patients with Rheumatoid Arthritis: The RRRR Study, a Randomised Controlled Trial’ by Tanaka et Al. Ann. Rheum. Dis..

[127] Colman R.J., Portocarrero-Castillo A., Chona D., Hellmann J., Minar P., Rosen M.J. (2021). Favorable Outcomes and Anti-TNF Durability After Addition of an Immunomodulator for Anti-Drug Antibodies in Pediatric IBD Patients. Inflamm. Bowel Dis..

[128] Singh S., Murad M.H., Fumery M., Sedano R., Jairath V., Panaccione R., Sandborn W.J., Ma C. (2021). Comparative Efficacy and Safety of Biologic Therapies for Moderate-to-Severe Crohn’s Disease: A Systematic Review and Network Meta-Analysis. Lancet Gastroenterol. Hepatol..

[129] Fiorino G., Danese S. (2016). Adalimumab and Azathioprine Combination Therapy for Crohn’s Disease: A Shining Diamond?. J. Crohn’s Colitis.

[130] van Schouwenburg P.A., Krieckaert C.L., Rispens T., Aarden L., Wolbink G.J., Wouters D. (2013). Long-Term Measurement of Anti-Adalimumab Using PH-Shift-Anti-Idiotype Antigen Binding Test Shows Predictive Value and Transient Antibody Formation. Ann. Rheum. Dis..

[131] Steenholdt C., Al-khalaf M., Brynskov J., Bendtzen K., Thomsen O., Ainsworth M.A. (2012). Clinical Implications of Variations in Anti-Infliximab Antibody Levels in Patients with Inflammatory Bowel Disease. Inflamm. Bowel Dis..

[132] Cludts I., Spinelli F.R., Morello F., Hockley J., Valesini G., Wadhwa M. (2018). Reprint of “Anti-Therapeutic Antibodies and Their Clinical Impact in Patients Treated with the TNF Antagonist Adalimumab”. Cytokine.

[133] Chung E.S., Packer M., Lo K.H., Fasanmade A.A., Willerson J.T. (2003). Randomized, Double-Blind, Placebo-Controlled, Pilot Trial of Infliximab, a Chimeric Monoclonal Antibody to Tumor Necrosis Factor-α, in Patients With Moderate-to-Severe Heart Failure. Circulation.

[134] Mann D.L., McMurray J.J.V., Packer M., Swedberg K., Borer J.S., Colucci W.S., Djian J., Drexler H., Feldman A., Kober L. (2004). Targeted Anticytokine Therapy in Patients With Chronic Heart Failure. Circulation.

[135] Keating E., Kelleher T.B., Lahiff C. (2020). De Novo Anti-TNF-α-Induced Congestive Heart Failure in a Patient With Turner Syndrome and Crohn’s Disease. Inflamm. Bowel Dis..

[136] Smeele H.T.W., Röder E., Mulders A.G.M.G.J., Steegers E.A.P., Dolhain R.J.E.M. (2022). Tumour Necrosis Factor Inhibitor Use during Pregnancy Is Associated with Increased Birth Weight of Rheumatoid Arthritis Patients’ Offspring. Ann. Rheum. Dis..

[137] Beltagy A., Aghamajidi A., Trespidi L., Ossola W., Meroni P.L. (2021). Biologics During Pregnancy and Breastfeeding Among Women With Rheumatic Diseases: Safety Clinical Evidence on the Road. Front. Pharmacol..

[138] Julsgaard M., Christensen L.A., Gibson P.R., Gearry R.B., Fallingborg J., Hvas C.L., Bibby B.M., Uldbjerg N., Connell W.R., Rosella O. (2016). Concentrations of Adalimumab and Infliximab in Mothers and Newborns, and Effects on Infection. Gastroenterology.

[139] Gupta R., Levin E., Wu J.J., Koo J., Liao W. (2014). An Update on Drug–Drug Interactions with Biologics for the Treatment of Moderate-to-Severe Psoriasis. J. Dermatolog. Treat..

[140] (2005). Etanercept plus Standard Therapy for Wegener’s Granulomatosis. N. Engl. J. Med..

[141] Bradley B.T., Maioli H., Johnston R., Chaudhry I., Fink S.L., Xu H., Najafian B., Deutsch G., Lacy J.M., Williams T. (2020). Histopathology and Ultrastructural Findings of Fatal COVID-19 Infections in Washington State: A Case Series. Lancet.

[142] Zou X., Chen K., Zou J., Han P., Hao J., Han Z. (2020). Single-Cell RNA-Seq Data Analysis on the Receptor ACE2 Expression Reveals the Potential Risk of Different Human Organs Vulnerable to 2019-NCoV Infection. Front. Med..

[143] Lopes-Pacheco M., Silva P.L., Cruz F.F., Battaglini D., Robba C., Pelosi P., Morales M.M., Caruso Neves C., Rocco P.R.M. (2021). Pathogenesis of Multiple Organ Injury in COVID-19 and Potential Therapeutic Strategies. Front. Physiol..

[144] Soy M., Keser G., Atagündüz P., Tabak F., Atagündüz I., Kayhan S. (2020). Cytokine Storm in COVID-19: Pathogenesis and Overview of Anti-Inflammatory Agents Used in Treatment. Clin. Rheumatol..

[145] Gianfrancesco M., Hyrich K.L., Al-Adely S., Carmona L., Danila M.I., Gossec L., Izadi Z., Jacobsohn L., Katz P., Lawson-Tovey S. (2020). Characteristics Associated with Hospitalisation for COVID-19 in People with Rheumatic Disease: Data from the COVID-19 Global Rheumatology Alliance Physician-Reported Registry. Ann. Rheum. Dis..

[146] Ungaro R.C., Brenner E.J., Agrawal M., Zhang X., Kappelman M.D., Colombel J.-F., Gearry R.B., Kaplan G.G., Kissous-Hunt M., Lewis J.D. (2022). Impact of Medications on COVID-19 Outcomes in Inflammatory Bowel Disease: Analysis of More Than 6000 Patients From an International Registry. Gastroenterology.

[147] SECURE-IBD (2022). Surveillance Epidemiology of Coronavirus under Research Exclusion. https://covidibd.org/current-data/.

[148] Sparks J.A., Wallace Z.S., Seet A.M., Gianfrancesco M.A., Izadi Z., Hyrich K.L., Strangfeld A., Gossec L., Carmona L., Mateus E.F. (2021). Associations of Baseline Use of Biologic or Targeted Synthetic DMARDs with COVID-19 Severity in Rheumatoid Arthritis: Results from the COVID-19 Global Rheumatology Alliance Physician Registry. Ann. Rheum. Dis..

[149] Lin S., Lau L.H., Chanchlani N., Kennedy N.A., Ng S.C. (2022). Recent Advances in Clinical Practice: Management of Inflammatory Bowel Disease during the COVID-19 Pandemic. Gut.

[150] Edelman-Klapper H., Zittan E., Bar-Gil Shitrit A., Rabinowitz K.M., Goren I., Avni-Biron I., Ollech J.E., Lichtenstein L., Banai-Eran H., Yanai H. (2022). Lower Serologic Response to COVID-19 MRNA Vaccine in Patients With Inflammatory Bowel Diseases Treated With Anti-TNFα. Gastroenterology.

[151] Basile M.S., Ciurleo R., Bramanti A., Petralia M.C., Fagone P., Nicoletti F., Cavalli E. (2021). Cognitive Decline in Rheumatoid Arthritis: Insight into the Molecular Pathogenetic Mechanisms. Int. J. Mol. Sci..

[152] Moyse E., Krantic S., Djellouli N., Roger S., Angoulvant D., Debacq C., Leroy V., Fougere B., Aidoud A. (2022). Neuroinflammation: A Possible Link Between Chronic Vascular Disorders and Neurodegenerative Diseases. Front. Aging Neurosci..

[153] Müller N. (2018). Inflammation in Schizophrenia: Pathogenetic Aspects and Therapeutic Considerations. Schizophr. Bull..

[154] Bauer M.E., Teixeira A.L. (2019). Inflammation in Psychiatric Disorders: What Comes First?. Ann. N. Y. Acad. Sci..

[155] Beurel E., Toups M., Nemeroff C.B. (2020). The Bidirectional Relationship of Depression and Inflammation: Double Trouble. Neuron.

[156] Cunningham C. (2013). Microglia and Neurodegeneration: The Role of Systemic Inflammation. Glia.

[157] Galea I. (2021). The Blood–Brain Barrier in Systemic Infection and Inflammation. Cell. Mol. Immunol..

[158] Liu L., Liu J., Bao J., Bai Q., Wang G. (2020). Interaction of Microglia and Astrocytes in the Neurovascular Unit. Front. Immunol..

[159] Karthikeyan S., Dimick M.K., Fiksenbaum L., Jeong H., Birmaher B., Kennedy J.L., Lanctôt K., Levitt A.J., Miller G.E., Schaffer A. (2022). Inflammatory Markers, Brain-Derived Neurotrophic Factor, and the Symptomatic Course of Adolescent Bipolar Disorder: A Prospective Repeated-Measures Study. Brain. Behav. Immun..

[160] Enache D., Pariante C.M., Mondelli V. (2019). Markers of Central Inflammation in Major Depressive Disorder: A Systematic Review and Meta-Analysis of Studies Examining Cerebrospinal Fluid, Positron Emission Tomography and Post-Mortem Brain Tissue. Brain. Behav. Immun..

[161] Hidese S., Hattori K., Sasayama D., Tsumagari T., Miyakawa T., Matsumura R., Yokota Y., Ishida I., Matsuo J., Yoshida S. (2021). Cerebrospinal Fluid Inflammatory Cytokine Levels in Patients With Major Psychiatric Disorders: A Multiplex Immunoassay Study. Front. Pharmacol..

[162] Petralia M.C., Mazzon E., Fagone P., Basile M.S., Lenzo V., Quattropani M.C., Bendtzen K., Nicoletti F. (2020). Pathogenic Contribution of the Macrophage Migration Inhibitory Factor Family to Major Depressive Disorder and Emerging Tailored Therapeutic Approaches. J. Affect. Disord..

[163] Stosic-Grujicic S., Stojanovic I., Nicoletti F. (2009). MIF in Autoimmunity and Novel Therapeutic Approaches. Autoimmun. Rev..

[164] Das R., Emon M.P.Z., Shahriar M., Nahar Z., Islam S.M.A., Bhuiyan M.A., Islam S.N., Islam M.R. (2021). Higher Levels of Serum IL-1β and TNF-α Are Associated with an Increased Probability of Major Depressive Disorder. Psychiatry Res..

[165] Mooney J.J., Brady R.O. (2018). Lithium + Colchicine. J. Clin. Psychopharmacol..

[166] Kenis G., Maes M. (2002). Effects of Antidepressants on the Production of Cytokines. Int. J. Neuropsychopharmacol..

[167] Benedetti F., Poletti S., Hoogenboezem T.A., Mazza E., Ambrée O., de Wit H., Wijkhuijs A.J.M., Locatelli C., Bollettini I., Colombo C. (2016). Inflammatory Cytokines Influence Measures of White Matter Integrity in Bipolar Disorder. J. Affect. Disord..

[168] Brymer K.J., Fenton E.Y., Kalynchuk L.E., Caruncho H.J. (2018). Peripheral Etanercept Administration Normalizes Behavior, Hippocampal Neurogenesis, and Hippocampal Reelin and GABAA Receptor Expression in a Preclinical Model of Depression. Front. Pharmacol..

[169] Alshammari M.A., Khan M.R., Majid Mahmood H., Alshehri A.O., Alasmari F.F., Alqahtani F.M., Alasmari A.F., Alsharari S.D., Alhossan A., Ahmad S.F. (2020). Systemic TNF-α Blockade Attenuates Anxiety and Depressive-like Behaviors in Db/Db Mice through Downregulation of Inflammatory Signaling in Peripheral Immune Cells. Saudi Pharm. J..

[170] Torres-Acosta N., O’Keefe J.H., O’Keefe E.L., Isaacson R., Small G. (2020). Therapeutic Potential of TNF-α Inhibition for Alzheimer’s Disease Prevention. J. Alzheimer’s Dis..

[171] Boufidou F., Nikolaou C. (2016). Anti-Inflammatory Medication as Adjunctive Antidepressive Treatment. Psychiatriki.

[172] Mansur R.B., Subramaniapillai M., Lee Y., Pan Z., Carmona N.E., Shekotikhina M., Iacobucci M., Rodrigues N., Nasri F., Rashidian H. (2020). Leptin Mediates Improvements in Cognitive Function Following Treatment with Infliximab in Adults with Bipolar Depression. Psychoneuroendocrinology.

[173] Mansur R.B., Subramaniapillai M., Lee Y., Pan Z., Carmona N.E., Shekotikhina M., Iacobucci M., Rodrigues N., Nasri F., Rosenblat J.D. (2021). Effects of Infliximab on Brain Neurochemistry of Adults with Bipolar Depression. J. Affect. Disord..

[174] McIntyre R.S., Subramaniapillai M., Lee Y., Pan Z., Carmona N.E., Shekotikhina M., Rosenblat J.D., Brietzke E., Soczynska J.K., Cosgrove V.E. (2019). Efficacy of Adjunctive Infliximab vs Placebo in the Treatment of Adults with Bipolar I/II Depression. JAMA Psychiatry.

[175] Lee Y., Mansur R.B., Brietzke E., Carmona N.E., Subramaniapillai M., Pan Z., Shekotikhina M., Rosenblat J.D., Suppes T., Cosgrove V.E. (2020). Efficacy of Adjunctive Infliximab vs. Placebo in the Treatment of Anhedonia in Bipolar I/II Depression. Brain. Behav. Immun..

[176] Bavaresco D.V., Uggioni M.L.R., Ferraz S.D., Marques R.M.M., Simon C.S., Dagostin V.S., Grande A.J., da Rosa M.I. (2020). Efficacy of Infliximab in Treatment-Resistant Depression: A Systematic Review and Meta-Analysis. Pharmacol. Biochem. Behav..

[177] MacPherson K.P., Sompol P., Kannarkat G.T., Chang J., Sniffen L., Wildner M.E., Norris C.M., Tansey M.G. (2017). Peripheral Administration of the Soluble TNF Inhibitor XPro1595 Modifies Brain Immune Cell Profiles, Decreases Beta-Amyloid Plaque Load, and Rescues Impaired Long-Term Potentiation in 5xFAD Mice. Neurobiol. Dis..

[178] Shi J.-Q., Shen W., Chen J., Wang B.-R., Zhong L.-L., Zhu Y.-W., Zhu H.-Q., Zhang Q.-Q., Zhang Y.-D., Xu J. (2011). Anti-TNF-α Reduces Amyloid Plaques and Tau Phosphorylation and Induces CD11c-Positive Dendritic-like Cell in the APP/PS1 Transgenic Mouse Brains. Brain Res..

[179] Kim D.H., Choi S.-M., Jho J., Park M.-S., Kang J., Park S.J., Ryu J.H., Jo J., Kim H.H., Kim B.C. (2016). Infliximab Ameliorates AD-Associated Object Recognition Memory Impairment. Behav. Brain Res..

[180] Shi J.-Q., Wang B.-R., Jiang W.-W., Chen J., Zhu Y.-W., Zhong L.-L., Zhang Y.-D., Xu J. (2011). Cognitive improvement with intrathecal administration of infliximab in a woman with alzheimer’s disease. J. Am. Geriatr. Soc..

[181] Ou W., Ohno Y., Yang J., Chandrashekar D.V., Abdullah T., Sun J., Murphy R., Roules C., Jagadeesan N., Cribbs D.H. (2022). Efficacy and Safety of a Brain-Penetrant Biologic TNF-α Inhibitor in Aged APP/PS1 Mice. Pharmaceutics.

[182] Ou W., Yang J., Simanauskaite J., Choi M., Castellanos D.M., Chang R., Sun J., Jagadeesan N., Parfitt K.D., Cribbs D.H. (2021). Biologic TNF-α Inhibitors Reduce Microgliosis, Neuronal Loss, and Tau Phosphorylation in a Transgenic Mouse Model of Tauopathy. J. Neuroinflammation.

[183] Boado R.J., Lu J.Z., Hui E.K.-W., Lin H., Pardridge W.M. (2019). Bi-Functional IgG-Lysosomal Enzyme Fusion Proteins for Brain Drug Delivery. Sci. Rep..

[184] Boado R.J. (2022). IgG Fusion Proteins for Brain Delivery of Biologics via Blood–Brain Barrier Receptor-Mediated Transport. Pharmaceutics.

[185] Miola A., Dal Porto V., Meda N., Perini G., Solmi M., Sambataro F. (2022). Secondary Mania Induced by TNF-α Inhibitors: A Systematic Review. Psychiatry Clin. Neurosci..

[186] Bystrom J., Clanchy F.I., Taher T.E., Al-Bogami M.M., Muhammad H.A., Alzabin S., Mangat P., Jawad A.S., Williams R.O., Mageed R.A. (2017). Response to Treatment with TNFα Inhibitors in Rheumatoid Arthritis Is Associated with High Levels of GM-CSF and GM-CSF+ T Lymphocytes. Clin. Rev. Allergy Immunol..

[187] Nguyen M.V.C., Baillet A., Romand X., Trocmé C., Courtier A., Marotte H., Thomas T., Soubrier M., Miossec P., Tébib J. (2019). Prealbumin, Platelet Factor 4 and S100A12 Combination at Baseline Predicts Good Response to TNF Alpha Inhibitors in Rheumatoid Arthritis. Jt. Bone Spine.

[188] Wijbrandts C.A., Dijkgraaf M.G.W., Kraan M.C., Vinkenoog M., Smeets T.J., Dinant H., Vos K., Lems W.F., Wolbink G.J., Sijpkens D. (2008). The Clinical Response to Infliximab in Rheumatoid Arthritis Is in Part Dependent on Pretreatment Tumour Necrosis Factor Expression in the Synovium. Ann. Rheum. Dis..

[189] Gaujoux R., Starosvetsky E., Maimon N., Vallania F., Bar-Yoseph H., Pressman S., Weisshof R., Goren I., Rabinowitz K., Waterman M. (2019). Cell-Centred Meta-Analysis Reveals Baseline Predictors of Anti-TNFα Non-Response in Biopsy and Blood of Patients with IBD. Gut.

[190] Kwak M.S., Cha J.M., Jeon J.W., Yoon J.Y., Park S.B. (2022). Uncovering Novel Pre-Treatment Molecular Biomarkers for Anti-TNF Therapeutic Response in Patients with Crohn’s Disease. J. Funct. Biomater..

[191] Magee C., Jethwa H., FitzGerald O.M., Jadon D.R. (2021). Biomarkers Predictive of Treatment Response in Psoriasis and Psoriatic Arthritis: A Systematic Review. Ther. Adv. Musculoskelet. Dis..

[192] Canet L.M., Cáliz R., Lupiañez C.B., Canhão H., Martinez M., Escudero A., Filipescu I., Segura-Catena J., Soto-Pino M.J., Ferrer M.A. (2015). Genetic Variants within Immune-Modulating Genes Influence the Risk of Developing Rheumatoid Arthritis and Anti-TNF Drug Response. Pharm. Genom..

[193] Sazonovs A., Kennedy N.A., Moutsianas L., Heap G.A., Rice D.L., Reppell M., Bewshea C.M., Chanchlani N., Walker G.J., Perry M.H. (2020). HLA-DQA1*05 Carriage Associated With Development of Anti-Drug Antibodies to Infliximab and Adalimumab in Patients With Crohn’s Disease. Gastroenterology.

